# Experimental or RCT research designs: a crisis of nomenclature in medical education

**Published:** 2012-09-30

**Authors:** Tyrone Donnon

**Affiliations:** University of Calgary, Calgary, Alberta, Canada

Medical education research draws on a history of quantification espoused by the physical sciences and more recently framed by theorists and researchers from the education and psychological disciplines of the late 19th to early 20th centuries. In the 1920s, the tradition of experimental research design in education achieved a heightened period of interest with the work of psychologists like Edward Thorndike (i.e., his research on learning processes led to the scientific foundation for modern educational psychology). The evolution of quantitative research in medical education stems from researchers’ interest in using a systematic empirical methodology to investigate and develop models, theories and hypotheses related to educational phenomena.

## True experiments in medical education

**Figure 1:**
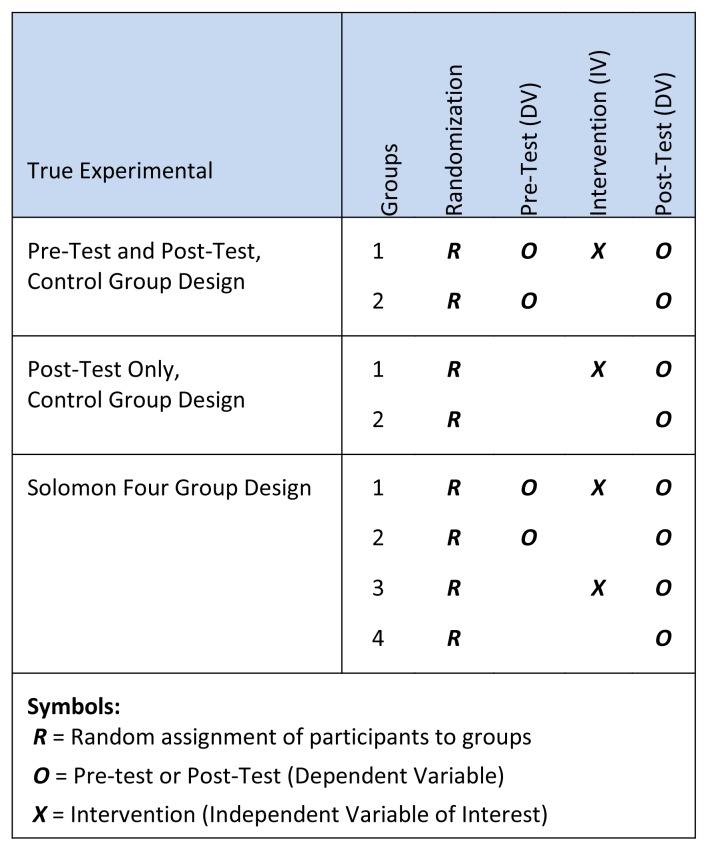
True experimental research designs.

In general, quantitative methods in medical education can be grouped into one of two sets of research designs: *observational* studies that focus on describing the situation, and *experimental* studies that investigate the effects related to a manipulated variable commonly referred to as the educational intervention. In 1965, Campbell and Stanley^1^ published the first book that defined experimental research designs into three general categories: *pre-*, *quasi-* and *true* (use of randomization). While the Solomon four group design^2^ is identified as being the optimal (but most impractical) true experimental research design, when possible educational researchers strive to achieve the *pre-test and post-test, control group design* (Figure 1)*.* By randomizing learners into the intervention group 1 or control group 2, the researcher strives to anticipate confounding factors that may influence the internal validity concerns related to doing quantitative studies in medical education. Nevertheless, it is important to understand that the use of a pre-test and post-test, control group design does not guarantee that other extraneous variables may not influence the reliability and validity of your findings. For example, in a study that investigated the use of cognitive imaging in the development of laparoscopic suturing skills, medical students were initially stratified by sex* (male or female) before being randomized into either the intervention or control groups.^3^ In this experimental study, the results of the use of cognitive imaging (i.e., a full week of mental rehearsal practice for the intervention group) was shown not to improve participants’ ability to complete a set of modified laparoscopic suturing tasks. There was found to be a statistically significant difference, however, on the students’ performance as a result of the extraneous variable sex (as a wealth of previous psychological research into spatial imaging has shown that males are better able to convert two-dimensional images, similar to what is seen on a monitor during laparoscopic surgery, into corresponding three-dimensional images and actions during the manipulation of objects within a closed endoscopic trainer).

## Randomized controlled trials and medical education?

The premise for randomization in both true experimental designs and clinical RCTs is an attempt by the researcher to control for all other potential influences (i.e., *independent variables* (IVs) that include general demographic characteristics of study participants or the manipulated variable of interest, and *dependent variables* (DVs) that provide the researcher with measures/assessments of change to participants’ intended outcomes. In medical education, these dependent measures can be provided by the participant in the form of achievement scores on written exams or changes in attitude through completion of self-reported surveys or questionnaires. Alternatively, observation of participants’ behaviours or performances can be obtained from examiners or evaluators using checklists and global rating scales.

In comparison, the randomized controlled trial (RCT) or randomized controlled clinical trial^4^ is a research design where people or, more specifically, patients are allocated randomly to receive one of any number of clinical treatments. The use of this nomenclature was established with Hill’s (1952) work on the description of clinical trials.^6^ In his description of RCTs, patients are randomly allocated into one or another group that are subjected to a special treatment (or treatments) and are compared, usually, to an administered placebo or a traditional approach to patient care on any number of clinical measures. In particular, Jadad’s 1998 book on *Randomised Controlled Trials* provides a comprehensive classification of RCTs that emphasizes the importance of this research design in meeting the specific needs of clinical research studies.^7^ For example, Phase 1, 2, 3 and 4 trials are specific terms used to define the study design approach for the introduction of a new treatment (typically a drug) where the initial Phase 1 studies confirm the safety of the treatment through animal testing as a first step to ensuring safety of the treatment with humans. Phase 2 studies begin the process of testing with small groups of real patients, assessing the efficacy of treatment modes of administration while monitoring safety effectiveness. Other examples of the usefulness of the RCT study designs in clinical research are the use of sample sizes where only one patient is selected for the trail (*N*-of-one trials) or where both patients and the treating doctors do not know who is receiving the treatment or placebo (double-blinded). While relevant for clinical research, such RCT research designs are impractical and impossible to expect from participants in medical education interventions (generally, the learners know the type of intervention they are receiving, and preventing them from reading or learning more about the process or content related to the study is unrealistic).

The major issue faced by researchers, however, is that, unlike clinical trials, it is impossible to blind teachers and students to the interventions provided while at the same time expecting participants (or the teachers) not to educate themselves during the study period. Correspondingly, most educational studies may demonstrate statistically significant changes to participants’ knowledge, skills and/or attitudes within a specific research time period and contextual setting, however, in most cases it is not reasonable to expect direct connections between an educational intervention provided in medical school, residency, or workshop, and what physicians will do in their actual practice.^8^

## Quantitative research in medical education

Experimental research designs allow the researcher to test hypotheses by investigating the cause and effect relationship between variables. An experimental study in medical education, however, is different from an observation study in one crucial way: the researcher is actively involved with the manipulation of the educational intervention or experimental variable – that independent variable of interest thought to make a difference to participants’ knowledge, skills and/or attitudes. In this regard, the researcher varies the intensity and duration of the intervention to study the influence an education initiative may have on the dependent variables of interest (e.g., enhanced understandings, performance mastery, professional values, and, at the very least, students’ satisfaction).

Medical education lies between two disciplines: education and medicine. As a field of study, this puts researchers in the precarious position of not being able to fund research projects simply because large funding agencies will only fund either medical sciences or social sciences/humanities (with the consequence of being rejected by both). Therefore, where the emphasis is on enhancing the educational components related to curriculum, teaching and assessment, the term *experimental* research designs are used to classify the levels of quantitative studies conducted in education. Unlike clinical research where specific trials are used to study the influence of drugs (placebos), clinical treatment, etc., to enhance patient outcomes, the focus of medical education research is to study the influence of educational interventions that lead to improved learner outcomes and, ultimately, better practitioners. The use of the term RCT to refer to true experimental research designs in medical education depreciates the hallmark of the randomized controlled trial in clinical research and introduces nomenclature confusion among researchers interested in educational and psychological research implications within a context and content that is medical only in name.

## Footnotes

* The correct term for determining the physiological characteristic of being a man (male) or woman (female) is “sex”, while the use of the term “gender” refers to the social-psychological construct used by society to define one’s connectedness to characteristics associated with being feminine, masculine, androgynous, amongst other categories.
